# Dynamical Patterning Modules, Biogeneric Materials, and the Evolution of Multicellular Plants

**DOI:** 10.3389/fpls.2018.00871

**Published:** 2018-07-16

**Authors:** Mariana Benítez, Valeria Hernández-Hernández, Stuart A. Newman, Karl J. Niklas

**Affiliations:** ^1^Centro de Ciencias de la Complejidad – Instituto de Ecología, Universidad Nacional Autónoma de México, Mexico City, Mexico; ^2^Laboratoire de Reproduction et Développement des Plantes, Université de Lyon, École Normale Supérieure de Lyon, Université Claude Bernard Lyon 1, Centre National de la Recherche Scientifique, Institut National de la Recherche Agronomique, Lyon, France; ^3^Department of Cell Biology and Anatomy, New York Medical College, Valhalla, NY, United States; ^4^Plant Biology Section, School of Integrative Plant Science, Cornell University, Ithaca, NY, United States

**Keywords:** plant evolution, plasmodesmata, algal evolution, convergent evolution, dynamical patterning modules

## Abstract

Comparative analyses of developmental processes across a broad spectrum of organisms are required to fully understand the mechanisms responsible for the major evolutionary transitions among eukaryotic photosynthetic lineages (defined here as the polyphyletic algae and the monophyletic land plants). The concepts of dynamical patterning modules (DPMs) and biogeneric materials provide a framework for studying developmental processes in the context of such comparative analyses. In the context of multicellularity, DPMs are defined as sets of conserved gene products and molecular networks, in conjunction with the physical morphogenetic and patterning processes they mobilize. A biogeneric material is defined as mesoscale matter with predictable morphogenetic capabilities that arise from complex cellular conglomerates. Using these concepts, we outline some of the main events and transitions in plant evolution, and describe the DPMs and biogeneric properties associated with and responsible for these transitions. We identify four primary DPMs that played critical roles in the evolution of multicellularity (i.e., the DPMs responsible for cell-to-cell adhesion, identifying the future cell wall, cell differentiation, and cell polarity). Three important conclusions emerge from a broad phyletic comparison: (1) DPMs have been achieved in different ways, even within the same clade (e.g., phycoplastic cell division in the Chlorophyta and phragmoplastic cell division in the Streptophyta), (2) DPMs had their origins in the co-option of molecular species present in the unicellular ancestors of multicellular plants, and (3) symplastic transport mediated by intercellular connections, particularly plasmodesmata, was critical for the evolution of complex multicellularity in plants.

## Introduction

**FIGURE 1:**
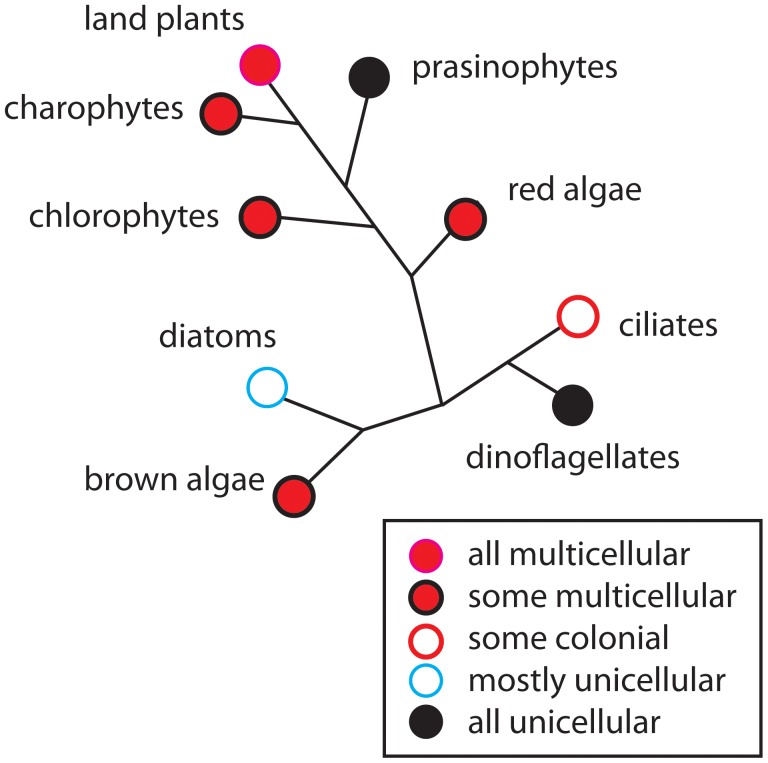
The occurrence of multicellularity shown on a highly redacted and unrooted phylogenetic diagram of the major groups of photosynthetic eukaryotes. Although some groups are entirely unicellular or multicellular (e.g., prasinophytes and the land plants, respectively), most contain a mixture of body plans such as the unicellular and colonial body plans (e.g., diatoms), or a mixture of the unicellular, colonial, and multicellular body plans (e.g., brown algae). In general, early-divergent persistent lineages are dominated by unicellular species (e.g., prasinophytes in the green algal clade), whereas later-divergent lineages contain a mixture of body plans (e.g., chlorophytes and charophytes). Species-rich, late-divergent persistent lineages tend to be exclusively multicellular (e.g., the land plants and metazoans).

The goal of this paper is to review the evolution of the multicellularity plant body plan within the conceptual framework of dynamic patterning modules (DPMs; Newman and Bhat, 2009; Newman et al., 2009; Newman, 2011), which provides a means of integrating physical and molecular-genetic aspects of developmental mechanisms. We have reviewed this topic previously (Hernández-Hernández et al., 2012; Niklas and Newman, 2013; Niklas, 2014). However, our focus here is on the DPMs involved in the establishment of body plan polarity and cell-tissue differentiation. As in our previous treatments of the topic, a broad comparative approach is adopted here because multicellularity has evolved multiple times among the various eukaryotic photosynthetic lineages (**Figure 1**). The exact number of times it has evolved in large part depends on how multicellularity is defined. If multicellularity is regarded as any transient or permanent aggregation of cells, it is estimated to have evolved independently at least 25 times (Grosberg and Strathmann, 2007). If more rigorous criteria are applied, as for example the requirement for intercellular communication and cooperation, multicellularity has evolved multiple times in the Actinobacteria, Myxobacteria, and Cyanobacteria, at least three times in the fungi (chytrids, ascomycetes, and basidiomycetes), six times among the polyphyletic algae (twice each in the red, brown, and green algae), but only once in the Animalia (Niklas and Newman, 2013; Niklas, 2014).

Regardless of how multicellularity is defined or how many times it evolved, the repeated independent evolution of multicellularity evokes many important but as yet unresolved questions. For example, are the developmental and morphological motifs involved in the transformation of unicellular organisms into multicellular ones adaptations to the exigencies of life, the result of weak selection pressures, or the predictable consequences of physico-genetic laws and processes? Do sets of “master genes” for multicellularity exist among most or all eukaryotic clades? Indeed, given its ubiquity among pro- and eukaryotic lineages, has multicellularity truly evolved independently among so many kinds of bacteria, fungi, algae, land plants, and animals, given the fact that all eukaryotes ultimately shared a last common ancestor?

The application of the framework of DPMs is particularly useful to address this last question because similar if not identical phenotypes can be achieved by the developmental mobilization of very dissimilar molecular systems or processes and because natural selection acts at the level of the phenotype and not at the level of the mechanisms that give rise to it. This dictum has been formalized by Newman and Bhat (2009), Newman et al. (2009), and Newman (2011) who have conceptualized the development and evolution of multicellular animals in the framework of DPMs each of which involves one or more sets of shared gene networks, their products, and the physical processes that relate to various types of matter. The importance of many of the physical processes involved in DPMs such as adhesion, cohesion, diffusion, activator–inhibitor dynamics, and viscoelasticity have long been recognized as important in development. Moreover, experimental research continues to demonstrate that the mechanical environment experienced by individual cells, tissues, and organs can alter gene expression patterns and thus cell fate specification (e.g., Swift et al., 2013).

Considering development and its evolution in terms of the DPM framework highlights the fact that the morphological motifs that are produced by physical processes evoked by specific molecules and pathways constitute a “pattern language” for configuring the basic body plans of multicellular animals and plants (Newman and Bhat, 2009; Newman et al., 2009; Hernández-Hernández et al., 2012; Niklas, 2014). These processes include mechanical forces resulting from the geometrical arrangements of mesoscale materials, irreversibility, the properties of network topologies and organization, and symmetry breaking. Importantly, many of the physical processes associated with DPMs are “generic” in that they are causally similar to the physical processes affecting the behavior of inorganic materials (Niklas, 1992; Niklas and Spatz, 2012). This congruence between the animate and inanimate world facilitated the rapid evolution of stereotypical generic morphologies once multicellularity was achieved in phyletically different groups of organisms, because there is ample evidence that some DPMs originated by means of the co-option of genes or gene regulatory networks (GRNs) present in ancestral unicellular organisms (Newman and Bhat, 2009; Newman et al., 2009).

**FIGURE 2:**
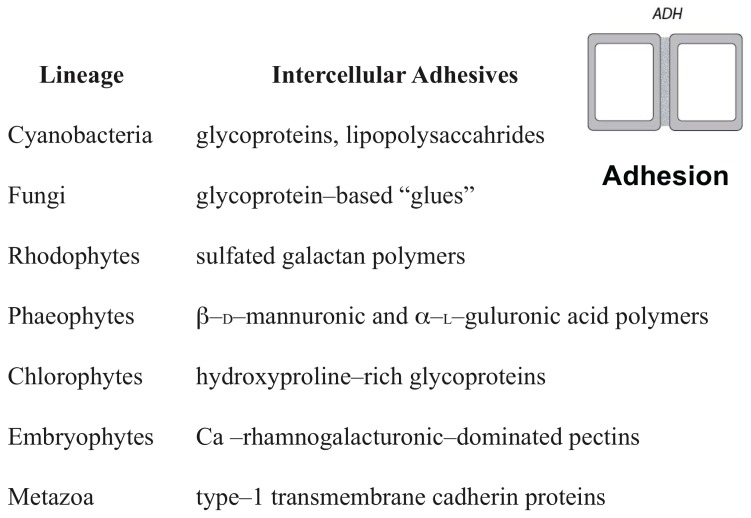
The different adhesives utilized by the cell-to-cell adhesive (ADH) dynamic patterning module among some of the major multicellular lineages.

Numerous examples of analogous DPMs operating across a broad spectrum of eukaryotic organisms can be given because of the many fundamental similarities existing among all eukaryotic cells (Wayne, 2009). For example, molecular pathways for the control of cell shape and polarity that evolved in unicellular organisms were mobilized by the novel protein Wnt in multicellular animals to mediate, via respective DPMs, lumen formation, and tissue elongation via convergent extension (reviewed in Newman, 2016b). Likewise, all eukaryotic cells have the capacity to produce extracellular (ergastic) polysaccharides and structural glycoproteins capable of self-assembly to create extracellular matrices containing interpenetrating polymeric networks of hydroxyproline-rich glycoproteins, e.g., collagen in animals and the extensin superfamily in numerous algal lineages and in the embryophytes (Ferris et al., 2001; Stanley et al., 2005) (**Figure 2**). These proteins manifest marked peptide periodicity, can form flexible rod-like molecules with repeat motifs (dominated by hydroxyproline) in helical configurations with arabinosyl/galactosyl side chains. This “superfamily” of intercellular adhesives operates among many unicellular organisms in gamete-to-gamete self-recognition and adhesion and the adhesion of cells to a substratum. It is very likely, therefore, that these “ancestral” adhesive capacities were co-opted to provide the cell-to-cell adhesives operating in many multicellular organisms, just as a wide array of microtubule-associated proteins in the algae, embryophytes, fungi, and metazoans (Gardiner, 2013) mediate related cell reshaping mechanisms utilized by DPMs in all these groups.

It is clear, however, that some of the DPMs operating in animals do not function in the various groups of algae, the land plants, and most fungi because of substantive differences among these lineages and clades (Meyerowitz, 2002; Newman and Niklas, 2018). Consider, for example, that the cells of animals are typically individually deformable and that during development they are free to move past one another in ways that permit differential adhesion, cortical tension, and other processes that permit the autonomous sorting and assembly of different tissues. In contrast, most plant and fungal cells possess a rigid cell wall that is firmly bound to the cell walls of adjoining cells. Likewise, plant signaling molecules acting as transcriptional modulators and determinants of tissue and cell fate can act intercellularly as well as intracellularly (see Cui et al., 2007; Urbanus et al., 2010; Garrett et al., 2012). This capacity, which is rare albeit not unknown in animal systems (Prochiantz, 2011), blurs the functional distinction of the GRNs affecting multi- versus single-cell differentiation.

**FIGURE 3:**
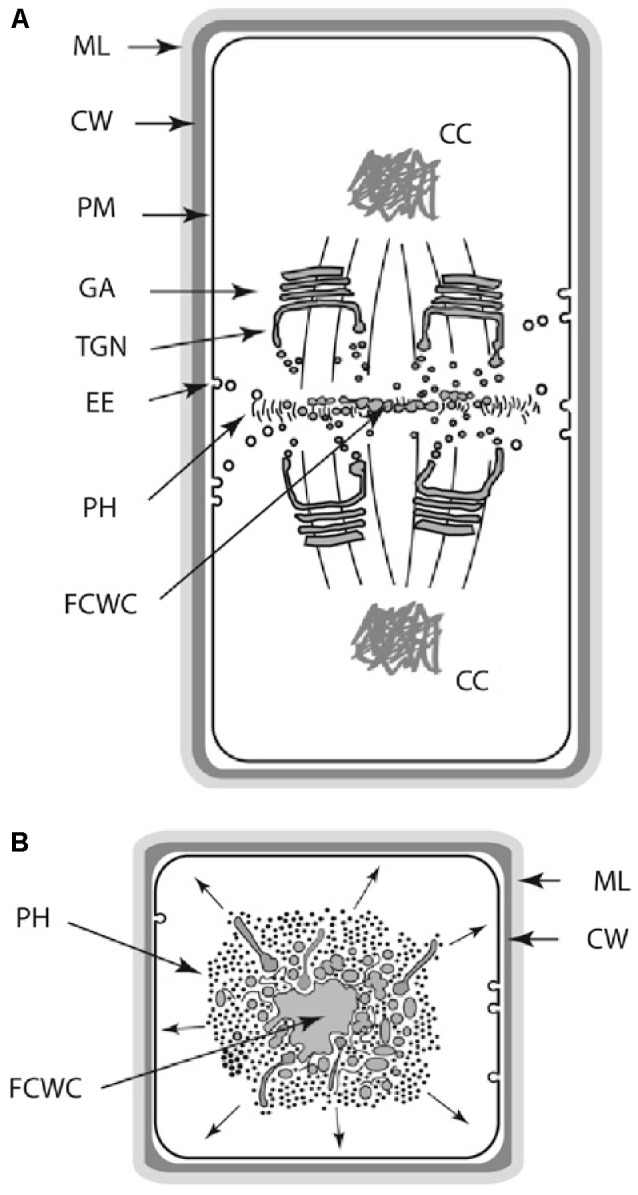
Schematic of the land plant phragmoplast (**A,B**: longitudinal and transverse views, respectively). AAC, apical actin cluster; AC, actin cable; AAEP, actin/ABPA endocytoic patches; CC, condensed chromatin; CW, cell wall; EE, endocytotic elements; FCWC, future cell wall components; GA, Golgi apparatus; ML, middle lamella; MT, microtubule; PH, phragmoplast; PM, plasma membrane; SE, post-Golgi sorting endosome; TGN, *trans*-Golgi network; VC, vesicle cluster.

Further, plant cell polarity involves the participation of PIN and PAN1 proteins in auxin polar and lateral transport and the regulation of metabolic fluxes by means of plasmodesmata. In contrast, animal cell polarity involves the participation of integrin, cadherin, and PAR or CDC42 proteins (Geldner, 2009; Dettmer and Friml, 2011; Zhang et al., 2012). Further, cell, tissue, and organ polarity within the multicellular plant body is maintained by a complex phytohormone transport system that involves the differential and sometimes transient positioning of auxin transporters proteins (Dettmer and Friml, 2011; Zhang et al., 2012), the establishment of mechanical heterogeneities within the apoplastic infrastructure (Kutschera and Niklas, 2007; Geldner, 2009; Peaucelle et al., 2015; Galletti et al., 2016; Majda et al., 2017), and the regulation of metabolic fluxes by means of plasmodesmata. Although analogies can be drawn between the establishment and maintenance of cellular and tissue polar domains in animals and plants, the mechanisms by which polarity is achieved are very different. For example, tight junctions between the apical and basolateral plasma membrane domains in animal epithelial cells provide barriers preventing the intramembrane diffusion of proteins and other macromolecules (Shin et al., 2006), whereas in plants a variety of phenolic compounds are used to maintain tissue polarity domains (Alassimone et al., 2010). As a final example of the differences between DPMs among the eukaryotic lineages, consider the manner in which cell wall materials are delivered and deposited during cell division. The mechanics of this developmental process differs substantively among the desmids and among different filamentous ascomycetes (Hall et al., 2008; Seiler and Justa-Schuch, 2010). It even differs within the monophyletic Chlorobionta, i.e., phycoplastic cell division in the Chlorophyta and phragmoplastic cell division in the Streptophyta (see Graham et al., 2009; Niklas, 2014) (**Figure 3**).

**FIGURE 4:**
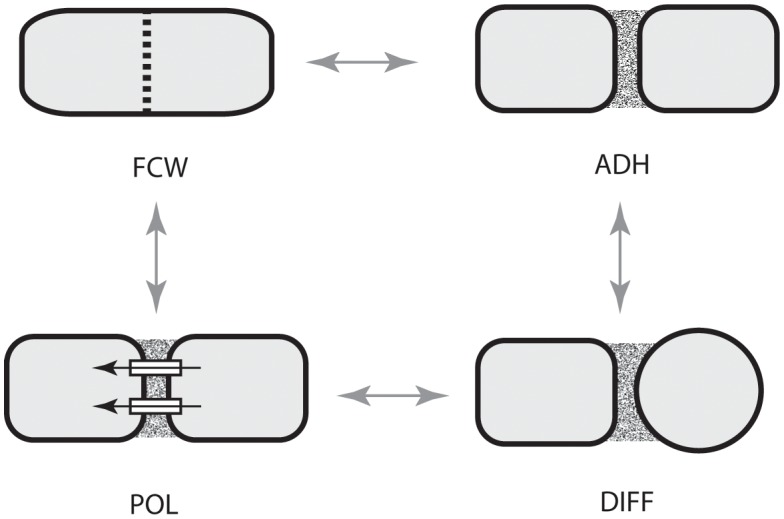
Paired dynamic patterning modules (indicated by arrows) that participate in the evolution of multicellularity. The acquisition of each of these modules is required for the evolution of multicellularity. These modules operate in pairs for organisms with cell walls because cell-to-cell adhesion is related to the location of a new cell wall and because intercellular communication operates in tandem with cell polarity. ADH, the capacity for cell-to-cell adhesion. DIF, the establishment of intercellular communication and cellular differentiation, FCW, the future cell wall module (establishes the location and orientation of the new cell wall), POL, the capacity for polar (preferential) intercellular transport. (Adapted from Hernández-Hernández et al., 2012.)

Focusing on the distinctive physico-genetic morphogenetic modalities of plants, Hernández-Hernández et al. (2012) identified six DPMs involved in critical embryophyte developmental processes. These DPMs are (1) the production of intercellular adhesives (ADH), (2) the manner in which the future cell wall is formed and oriented (FCW), (3) the establishment of intercellular communication and spatial-dependent patterns of differentiation (DIF), (4) the establishment of axial and lateral polarity (POL), (5) the formation of lateral appendages or “buds” (BUD), and (6) the formation of lateral, leaf-like structures (LLS). Hernández-Hernández et al. (2012) discussed all six of these DPMs with an emphasis on the first four (i.e., ADH, FCW, DIF, and POL), because cell division, cell-to-cell adhesion, intercellular communication, and polarity are essential for achieving simple multicellularity across all clades and because these four DPMs operate in a pairwise manner in many multicellular algae and fungi as well as in the land plants (**Figure 4**). Here, we emphasize DIF and POL because these are essential for achieving complex multicellularity (define here as “the condition in which some cells are not in direct contact with the external environment”), and present new evidence that the evolution of plasmodesmata played a critical role in the evolution of cell, tissue, and organ differentiation and polarity. We also identify the characteristic molecules and molecular networks, and when possible, the physical processes they mobilize for each of the four key modules.

## Biogeneric Materials and Dynamical Patterning Modules (DPMs)

Before proceeding to an exploration of the empirical evidence for the concepts that will be pursued, it is important to establish clear definitions for what is meant by biogeneric materials and DPMs, particularly since these concepts may not be familiar to some researchers and because clarity in definitions is essential for clarity in thinking.

Like all matter, living matter manifests inherent morphogenetic properties, and characteristic morphological motifs that are in part expressions of inviolable physical laws and principles (Newman, 2017; see also Niklas and Spatz, 2012; Niklas, 2017). This idea is familiar to those who study the physical sciences as is the notion that the operation of physical laws and principles is sensitive to scale (Niklas, 1994). On the macroscale, phenomena such as large-scale climate and oceanic systems generate fluidic patterns of various but discrete and recognizable morphologies. On the microscale, atoms and small molecules can be arranged and rearranged to form discrete molecules with well-defined chemical and physical properties. Living matter operates at an intermediate or *meso*scale. Non-living mesoscale materials are familiar as solids, which can be amorphous of crystalline, and liquids, which can form vortices and waves. Living matter exhibits many of these generic physical properties (Green and Batterman, 2017).

Although all living cells have mesoscale properties in common (e.g., their cell membranes and cytoplasm are rheologically similar), we focus here exclusively on multicellular matter. For example, all tissues behave as viscoelastic materials (i.e., they behave as a combination of a liquid and a solid). However, the extent to which a material manifests viscoelasticity depends on the presence and quantity of its rigid components (Niklas, 1989, 1992). With few exceptions (e.g., bone and cartilage), most animal tissues are highly viscoelastic owing to an absence of rigid cell walls. In contrast, all plant and fungal tissues behave as deformable cellular solids because of the presence of rigid cell wall solids (e.g., cellulose and chitin) (Niklas, 1989, 1992). Animal, plant, and fungal tissues have shared generic properties, but they differ in the degree to which they respond to physical stresses. Thus, the subunits – cells – of animal tissues can be independently mobile and rearrange with respect to one another, particularly during development, when the tissues are more liquid-like than they are in the mature organism, whereas, with the exception of intrusive growth, plant and fungal cells do not typically change their neighbors.

The generic physical properties of living matter lend predictability to the forms it can assume during development. Like non-living liquids, liquid crystals, and mixtures thereof, developing animal tissues can form immiscible layers, interior spaces, and undergo elongation (Forgacs and Newman, 2005). Like non-living deformable solids (undergoing, for example, accretion or melting), developing plant and fungal tissues can bud or branch (Fleury, 1999; Niklas and Spatz, 2012).

The liquid vs. solid nature of living tissues does not arise from the same subunit-subunit interactions that endow non-living materials with these properties. Instead of Brownian motion and the electronic weak attractive interactions among the molecular subunits of non-biological liquids, the cells in animal tissues move non-randomly by cytoskeletally generated forces and remain cohesive despite their translocation via transmembrane homophilic attachment proteins. In plant and fungal tissues, instead of the charge-based or covalent bonds of the atomic or molecular subunits of non-biological solids, the cells are cemented together by Ca^2+^-rhamnogalacturonanic-rich pectins, or members of the extensin superfamily of hydroxyproline-rich glycoproteins (Cannon et al., 2008; Lamport et al., 2011). For these and other reasons, the various viscoelastic and deformable solid materials that constitute living tissues have been termed “biogeneric” matter, in recognition of the predictability of their morphogenetic behavior and outcomes afforded by their generic properties, and the fact that these generic properties are dependent on evolved biological, rather than purely physical, effects (Newman, 2016a).

Another set of biogeneric properties that characterize living tissues, superimposed upon their identity as predominantly viscoelastic or non-deformable solid materials, is their *excitability*, that is, the ability to store energy and release it upon stimulation (Levine and Ben-Jacob, 2004; Sinha and Sridhar, 2015). Mechanically, chemically, and electrically excitable materials are not unknown in the non-living world (exemplified by loaded mousetraps, forest fires, and tunnel diodes) but they are uncommon. Multicellular systems are inevitably excitable, because their cellular subunits are biochemically, mechanically, and electrically active, the storage and controlled utilization of energy is intrinsic to all life (Lund, 1947).

During the development of multicellular organisms, communication among the cellular subunits can induce the spatiotemporal mobilization of mechanical, chemical, and electrical energy, leading to cellular pattern formation and morphogenesis. In animal embryos and organ primordia, this communication is generally short-range via extracellularly diffusible morphogens. However, mechanical and electrical fields can achieve nearly instantaneous long-range communication. In developing and remodeling plants and fungi, communication can be both intra- and intercellular and short and long-range. Like the biogeneric rheological and solid-state properties of animal, plant, and fungal tissues, the phenomena of excitability give rise to predictable morphological motifs – repetitive or fractal arrangements of ridges, appendages, venation patterns and cell types.

Dynamical patterning modules, defined above, are intrinsic to this “physico-genetic” account of the origin and evolution of multicellular life-forms (Newman, 2012). The DPM concept recognizes that the physical forces that shape tissues cannot be considered independently of the actual materials (cell collectivities and their molecules) that they act on. The activity of DPMs can be regulated in a given multicellular material (e.g., that characterizing the phylum to which a species belongs), leading to developmental transitions and phenotypic differences between members of a phylum. Insofar as the materials have biogeneric properties (as is the case with animal tissues, and to a large extent with plant and fungal tissues), specific DPMs will promote morphological outcomes familiar from the physics of non-living matter. In other cases, DPMs will mobilize physical forces to produce outcomes peculiar to varieties of living matter. Without exception, however, physics and genetics act together to effect morphological development.

The following sections will update earlier descriptions of DPMs in plant systems (Hernández-Hernández et al., 2012; Benítez and Hejatko, 2013; Niklas and Newman, 2013; Niklas et al., 2013; Niklas, 2014; Mora Van Cauwelaert et al., 2015) and attempt to assign evolutionary roles to them.

## Evolutionary Transitions in Eukaryotic Photosynthetic Lineages

The fossil record and contemporary molecular phylogenetic analyses indicate that the three major algal clades in which multicellularity evolved (i.e., the Streptopiles, Rhodophytes, and Chlorobionta) had independent evolutionary origins because of primary and secondary endosymbiotic events (reviewed by Kutschera and Niklas, 2005, 2008). Consequently, these three clades in tandem with the evolution of the land plants (from a green algal ancestor) can be viewed as independent “evolutionary experiments” that provide an opportunity to examine how the four DPMs (i.e., ADH, FCW, DIF, and POL) participated in achieving multicellularity in each case.

The significance of the four DPMs becomes apparent when they are placed in the context of a morphospace that identifies the major plant body plans and when their placement is juxtaposed with a series of phenotypic transformations predicted by multilevel selection theory for the evolutionary appearance of multicellularity regardless of the clade under consideration (Niklas, 2014). Following McGhee (1999), we define a morphospace as a depiction of all theoretically possible structural phenotypes within a specific group of organisms. The depiction is constructed using orthogonal axes, each of which represents a phenotypic character that has one or more character states, e.g., cellular aggregation: yes or no. The intersection of two or more such axes specifies a hypothetical or real phenotype defined by the variables or processes the participating and intersecting axes specify.

**FIGURE 5:**
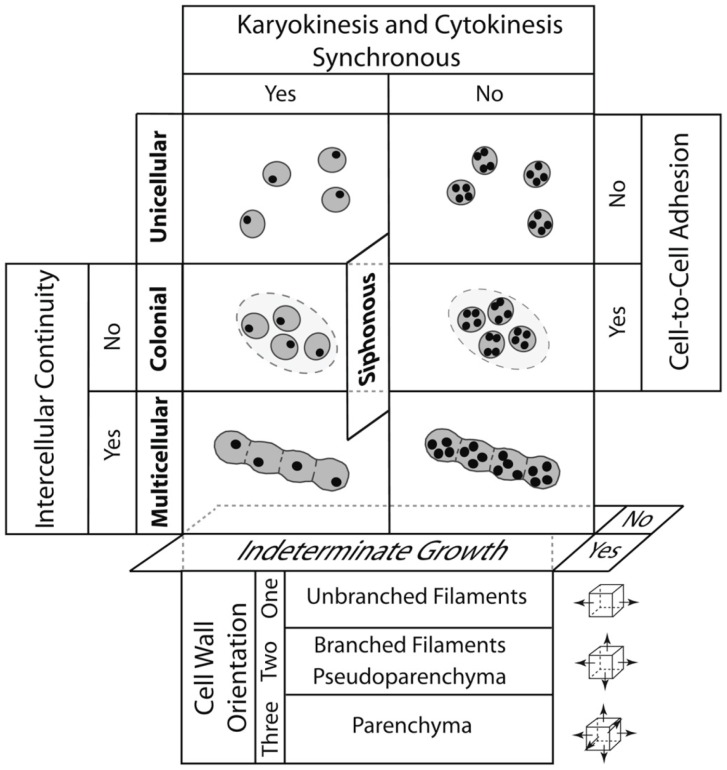
A morphospace for the four major plant body plans shown in bold (unicellular, siphonous/coenocytic, colonial, and multicellular) resulting from the intersection of five developmental processes: (1) whether cytokinesis and karyokinesis are synchronous, (2) whether cells remain aggregated after they divide, (3) whether symplastic continuity or some other form of intercellular communication is maintained among neighboring cells, and (4) whether individual cells continue to grow indefinitely in size. Note that the siphonous/coenocytic body plan may evolve from a unicellular or a multicellular progenitor. The lower panels deal with the plane of cell division (depicted by small cubes and arrows shown to the right) to yield unbranched and branched filaments, pseudoparenchyma, and parenchyma (found in the plants) and the localization of cellular division. The operation of the four DPMs reviewed in the article is summarized in **Figure 7**. (Adapted from Niklas, 2000, 2014).

Niklas (2000, 2014) constructed such a morphospace for all photosynthetic eukaryotes using four characters, each of which has two character states in the form of a question, i.e., (1) are cytokinesis and karyokinesis synchronous?; (2) do cells remain attached after cellular division?; (3) is symplastic or some other form of intercellular communication established and maintain among adjoining cells?; and (4) do individual cells continue to grow indefinitely in size? This simple morphospace identifies four plant body plans that can be either uninucleate or multinucleate, i.e., the unicellular, siphonous/coenocytic, colonial, and multicellular body plan (**Figure 5**). The different tissue constructs of the multicellular plant body can be identified also by adding a fifth axis that specifies the orientation of cell division with respect to the body axis. With the addition of this axis, three tissue constructs are identified, i.e., the unbranched filament (when cell division is restricted to one plane of reference), the branched filament and the pseudoparenchymatous tissue construct (when cell division occurs in two planes of reference and when branched filaments interweave, respectively), and the parenchymatous tissue construct (when cell division occurs in three planes of cell division).

The primary literature dealing with the algae (e.g., Graham et al., 2009) reveals that all four plant body plans (as well as the three tissue constructs) have evolved multiple times in the Stramenopiles, Rhodophytes, and Chlorobionta. This convergence reveals the evolutionary significance of the four DPMs that are the focus of our review, i.e., FCW, ADH, DIF, and POL (**Figure 4**). Specifically, ADH is required for the colonial and multicellular body plans; FCW is involved in whether cyto- and karyokinesis are synchronous and whether the tissue construction of a multicellular plant is filamentous (unbranched or branched) or parenchymatous, although how the FCW is determined remains problematic, even for the well studied land plants (Schaefer et al., 2017); and DIF and POL are required for intercellular cooperation and cellular specialization.

These four DPMs also help to identify evolutionary trends in the establishment of multicellularity predicted by multilevel selection theory (Folse and Roughgarden, 2010; Niklas and Newman, 2013; Niklas, 2014). This theory recognizes the unicellular organism as the ancestral state in each of the multicellular lineages or clades, and it identifies the colonial body plan as transitional to the multicellular body plan. Therefore, when multilevel selection theory is applied to the evolution of multicellularity, it identifies a “unicellular => colonial => multicellular” body plan transformation series (regardless of the type of organism) in which the participation of ADH, FCW, DIF, and POL are collectively required to establish and maintain a colonial body plan and to subsequently coordinate and specify the intercellular activities within an integrated multicellular body plan whose complexity exceeds simple dyatic interactions among conjoined cells, tissues, and organs.

The transformation series among the different genera and species of the volvocine algal lineage is consistent with the aforementioned multilevel selection theory’s predicted unicellular => colonial => multicellular transformation series (Bonner, 2000; Kirk, 2005; Herron and Michod, 2008; Niklas, 2014). The ancestral volvocine body plan undoubtedly possessed a unicellular organism that was morphologically and physiologically like *Chlamydomonas*. The transformation of this unicellular organism into a colonial organism is posited to have involved the modification of the ancestral cell wall into an extracellular adhesive matrix seen in the Tetrabaenaceae => Goniaceae => Volvocaceae transformation series (Kirk, 2005; see also Graham et al., 2009), which is consistent with what is known about the biochemistry of this ergastic material (Sumper and Hallmann, 1998). Subsequent evolutionary modifications exemplified by a hypothetical Goniaceae => Volvocaceae transformation series are predicted to have produced life-forms ranging from simple colonial aggregates (e.g., *Tetrabaena socialis*) to more colonies with asymmetric cell division, to multicellular organisms with a germ-soma division of labor (e.g., *Volvox carteri*) (Kirk, 2005; Herron and Michod, 2008). It is worth noting that in the case of multicellular volvocine algae, the cytoplasmic bridges that interconnect each cell to its neighbors have multiple functionalities. These bridges participate in the mechanics of a unique form of kinesin-driven inversion, and they provide avenues for the metabolic transport of nutrients to developing reproductive structures, called gonidia (Hoops et al., 2000). In this sense, these bridges are analogous to land plant plasmodesmata, although their apertures are much wider than those of the latter (∼200 nm in diameter; Green et al., 1981). Curiously, in some volvocines, these bridges are developmentally severed and thereby provide an interesting example of a multicellular-to-colonial transformation series.

## DPMs and Biogeneric Properties Involved in Plant Development and Evolutionary Transitions

As noted previously, we proposed a set of DPMs associated with key plant developmental events and specified some of the physical and molecular components of these modules (Hernández-Hernández et al., 2012). After reviewing the phyletic distribution of the molecular elements of the DPMs, we hypothesized that these modules originated from the co-option of cell-molecular mechanisms related to single-cell functions in the unicellular ancestors of the major algal clades and the land plant lineages that mobilized, in the multicellular context, novel physical processes. One of our central conclusions is that once development is set into operation, much of it becomes self-organizing due to the mobilization of DPMs and biogeneric properties. This view contrasts with the hypothesis that land plant diversification resulted mainly from the expansion of particular gene families (e.g., Vergara-Silva et al., 2000; Zažímalová et al., 2010). Certainly, while these molecules are central for plant development and, most probably also for plant evolution, we argued that the notion that diversification of certain gene families or molecular classes can be the main cause of morphological evolution is insufficient. Additionally, we suggested that the combination of different DPMs at different places and developmental stages may help understand the generation of the basic features of the multicellular plant body plan. We argued further that plant development has evolved into processes that occur in a physical medium that is dynamic over large scales, utilizing inherently multicellular systems of multifunctional hormones/morphogens/transcription factors that are unrestricted by cell boundaries in many of their functions. Under such conditions, the origin and mechanisms behind plant extraordinary plasticity becomes less enigmatic (Hernández-Hernández et al., 2012).

### The Spatially Dependent Differentiation (DIF) DPM and its Role in the Evolution of Multicellular Plants and Vascularization

**FIGURE 6:**
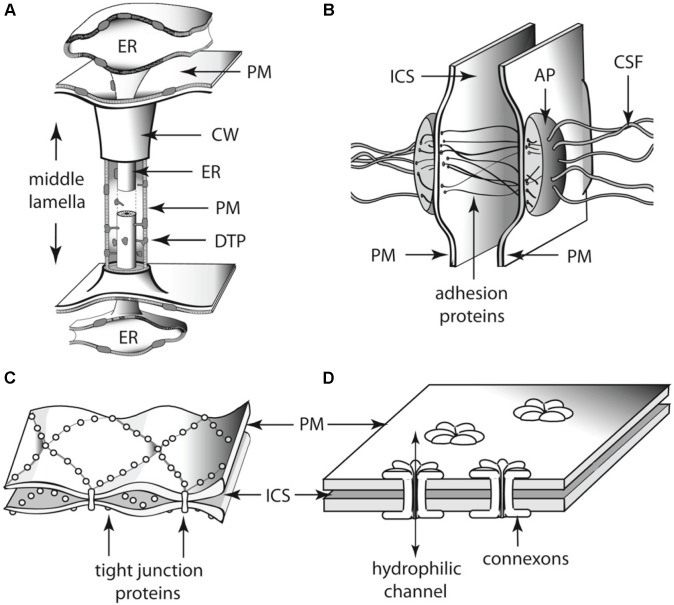
Schematics of the diversity of intercellular communication among adjoining **(A)** land plant and **(B–D)** animal cells. Each of these cell-to-cell linkages participates in the establishment of cell polarity as well as physiological communication among adjoining cells. Thus, each represents an analogous evolutionary innovation for two of the essential features of multicellularity. **(A)** Plasmodesma. **(B)** Desmosome. **(C)** Tight junctions. **(D)** Gap junctions. AP, attachment plaque (plakoglobins); CSF, cytoskeletal filaments (keratin); CW, cell wall; DTP, desmotubular proteins; ER, endoplasmic reticulum; ICS, intercellular space; PM, plasma membrane.

As groups of cells adhered to each other, some physical constraints were imposed on the transport of nutrients and signaling molecules. Multicellular aggregates eventually evolved a division of labor (Niklas, 2000; Kirk, 2005; Knoll, 2011) that required cell fate specification mechanisms (Niklas et al., 2014), and the ability of cells to coordinate their metabolism, patterns of cell growth, and the activity of molecular networks. In almost every multicellular aggregate two possible mechanisms for the exchange of nutrients and signaling molecules exist: indirect and direct transport (Beaumont, 2009). The first case requires some cells to secrete nutrients to the external environment and other cells to take them up (Beaumont, 2009). In contrast, for direct cell-to-cell transport, the presence of transmembrane connections is required (Niklas, 2000; Knoll, 2011). During the course of evolution, intercellular connections evolved independently in multicellular lineages to respond to the biophysical challenges that multicellularity imposed (**Figure 6**). For example, animals have gap junctions, whereas the cells of plants and fungi, respectively, developed plasmodesmata and septa pores, respectively (Bloemendal and Kück, 2013). Here, we discuss the role of plasmodesmata-mediated transport in the coordination of cell type specification during plant development and how this could have been a prerequisite for the transitions from unicellular to multicellular (and from non-vascular to vascular) plants. We will also briefly review the phylogenetic data concerning the evolution of plasmodesmatal structure.

Given that plant cells are surrounded by a rigid cell wall, they rely on the transport of signaling molecules for the establishment of cell type patterns. As we discussed in previous work (Hernández-Hernández et al., 2012), the spatially dependent differentiation (DIF) DPM is composed of the plant intercellular channels, called plasmodesmata, and the biogeneric properties they mobilize, viz., passive diffusion, lateral inhibition, and reaction–diffusion. Plants manifest a precise spatiotemporal control on the aperture (also called permeability) of plasmodesmata channels and can control the direction of plasmodesmata-mediated fluxes to create molecular concentration gradients (Sager and Lee, 2014). Using different techniques, Christensen et al. (2009) detected unidirectional transport of fluorescent probes from the basal epidermal cells into the apical cells of trichomes in the leaves of tobacco, *Nicotiana tabacum*. The probes were observed to move freely among trichome cells in both directions, but they were prevented from migrating in the opposite direction into subtending cells. Although the authors did not conclusively prove that this unidirectional flow depends on the aperture of plasmodesmata channels, they found that treatments with sodium azide, a metabolic inhibitor that alters plasmodesmata permeability, could reverse the direction of unidirectional flow from epidermal to trichome cells. A unidirectional transport of the photoconvertible dye Dendra2 was also observed in *Physcomitrella patens* (Kitagawa and Fujita, 2013), which indicted that this phenomenology is likely very ancient and thus of wide occurrence among the land plants. With the help of a controlled intercellular transport, plants can then modulate diffusion of signaling molecules in specific ways to generate or at least modulate patterns of cell type specification.

At the same time, the regulation of plasmodesmata permeability can generate morphogen gradients. Plasmodesmata aperture is regulated by the deposition and degradation of callose within the cell walls through which plasmodesmata pass (De Storme and Geelen, 2014). The turnover of callose is achieved by the participation of several families of proteins among which the GLUCAN SYNTHASE LIKE (GSL) proteins and β-glucanases, respectively, synthesize and degrade callose (Ruan et al., 2004; Guseman et al., 2010; De Storme and Geelen, 2014). Further, genetic and chemical experiments have correlated the amount of callose at plasmodesmatal sites with the genetic expression of GSLs and β-glucanases, and the intercellular migration of molecules in several plant systems (Ruan et al., 2004; Guseman et al., 2010; Vatén et al., 2011; Benitez-Alfonso et al., 2013; Han et al., 2014). For example, in hypocotyls of *Arabidopsis* seedlings, it was demonstrated that the reduced callose deposition at plasmodesmata, resulting from an inducible knock down mutation of the *GLUCAN SYNTHASE LIKE 8 (GSL8)* gene, had an enhanced diffusion of auxin (Han et al., 2014). Consequently, the loss of asymmetric auxin distribution prevented the differential cell elongation between the shaded and illuminated parts of the hypocotyl that is required for the phototropic response (Han et al., 2014). Based on these and other observations, Han et al. (2014) concluded that plasmodesmata closure is necessary to prevent auxin diffusion in *Arabidopsis* and to generate concentration gradients. In a similar way, it has been proposed that the main mechanism to establish auxin gradients in mosses such as *P. patens* is through plasmodesmata-mediated transport (Brunkard and Zambryski, 2017). Therefore, it seems likely that the regulation of plasmodesmata permeability has been key for land plants to establish concentration gradients of morphogens that coordinate developmental dynamics. However, it is important to note that neither plasmodesmata nor multicellularity are required to achieve morphological complexity. This is evident from siphonous (coenocytic) algae such as the marine green alga *Caulerpa*. A recent intracellular transcriptomic atlas of this organism reveals that the acropetal transcript distribution conforms roughly to a transcription-to-translation pattern without the presence of internal cell walls (Ranjan et al., 2015; see also Menzel, 1996).

Cell type specification that depends on the intercellular transport of transcription factors is also accompanied by the closure of plasmodesmata to actuate a lateral inhibition mechanism. For example, the *chor* mutant of *Arabidopsis*, which encodes a putative GSL8 protein, results in a significant increase in the number of stomatal lineage cells (Guseman et al., 2010). Further, in the epidermal cells of leaves, the expression of the SPEECHLESS (SPCH) transcription factor, which specifies the initiation of the stomatal lineage, is restricted to the meristemoid mother cells of stomata after asymmetric division (Pillitteri and Dong, 2013). The *gsl8* mutant has a lower amount of callose deposition resulting in the leakage of SPCH between epidermal cells that, in turn, results in abnormal stomata clusters (Guseman et al., 2010). By preventing the intercellular migration of SPCH, plasmodesmata inhibit the cells surrounding meristemoids to differentiate into the stomata lineage and thus regulate the spacing of stomata in the epidermis of leaves. This demonstrates that the plasmodesmata aperture is necessary for the specification of cell identities by virtue of regulating lateral inhibition.

The non-cellular autonomous signaling mediated by symplasmic transport is a key mechanism to establish patterns of cell specification required for the development of vascular tissues. For example, in the root of *Arabidopsis* the transcription factor SHORT ROOT (SHR) moves from the stele into the cells within the quiescent center and the endodermis where it turns on the production of miRNA165/6 (Carlsbecker et al., 2010). The miRNA165/6 then moves back to the stele where it degrades the homeodomain leucine zipper PHABULOSA (PHB), which is necessary for the radial patterning of the xylem tissue and the pericycle (Carlsbecker et al., 2010). Mutations of the CALLOSE SYNTHASE GLUCAN LIKE 3/GLUCAN SYNTHASE LIKE 12 (CALS3/GSL12) gene, which the product of degrades callose, results in an increased callose deposition (Vatén et al., 2011). In these mutants, the signal of pSHR:SHR:GFP in the endodermis relative to that of the stele is decreased when compared with the wild type. This observation is consistent with the hypothesis that callose deposition prevents symplasmic transport (Vatén et al., 2011). Because of the downregulated symplastic transport, protoxylem cell identity was disrupted and metaxylem cells were ectopically expressed in the location of protoxylem cells (Vatén et al., 2011). In this manner, it is possible that plasmodesmata-mediated transport may have also driven the development of specialized cells and tissues by means of the spatiotemporal differential transport of nutrients.

Finally, it has become increasingly clear that the manner in which plasmodesmata are distributed within the multicellular plant body compartmentalizes this body into symplastic domains that can take on different functionalities by virtue of either sequestering aspects of metabolic activity, as for example during the dormancy of terminal tree buds (Tylewicz et al., 2018) or facilitating specific avenues of symplastic translocation, as for example the movement of mRNA within the phloem (Xoconostle-Cazares et al., 1999). When seen in this manner, the multicellular plant body plan is actually a continuous symplast incompletely partitioned by a continuous apoplast created by an infrastructure of perforated cell walls (Niklas and Kaplan, 1991).

Indeed, all the available evidence demonstrates the importance of plasmodesmata-mediated transport for plant development (Sager and Lee, 2014). Plasmodesmata seem to have appeared independently several times in the plant kingdom. Intercellular connections very similar to the plasmodesmata of land plants have been found in the multicellular species of the green, red, and brown algae (e.g., Cook et al., 1997, 1999; Raven, 1997). As in the case of the land plants, the plasmodesmata of the green alga *Bulbochaete hiloensis* are modulated during ontogeny in a manner that differentially limits intercellular transport and separates cellular domains into different functional identities (Fraser and Gunning, 1969; Kwiatkowska, 1999).

Some features of plasmodesmata seem to have evolved after the Chlorophyte–Streptophyte divergence. For example, there is some evidence that the encapsulation of the endoplasmic reticulum (ER) within the plasmodesmatal channel is unique to the land plants. A close examination of plasmodesmata structure in the charophycean alga *Chara zeylanica* and in three putative early divergent bryophytes (the liverwort *Monoclea gottschei*, the hornwort *Notothylas orbicularis*, and the moss *Sphagnum fimbriatum*) reveals that in contrast to *C. zeylanica*, all three bryophytes have encased ER (Cook et al., 1997). The ER lumen serves as another pathway for intercellular transport making plasmodesmata transport more complex (Guenoune-Gelbart et al., 2008). The more complex plasmodesmata with internal ER of the land plants are present in some green algae, such as *Uronema* and *Aphanochaete* (Chlorophyceae) (Floyd et al., 1971; Stewart et al., 1973), and in some Laminariales brown algae (Marchant, 1976; Sideman and Scheirer, 1977). However, the movement of molecules through the lumen or the ER of these plasmodesmata has not been yet demonstrated for these algae. Based on these observations, it is reasonable to conclude that plasmodesmata lacking encased ER evolved first and that the encapsulation of ER is an evolutionarily derived feature that was present in the green algal ancestor of the land plants, well before bryophytes diverged (Lucas et al., 1993; Cook et al., 1997).

Given that the land plants are more complex than their algal ancestors because of the presence of specialized cell types and tissues for nutrient transport, we speculate that the increased complexity of multicellular plants is associated with the evolution of structurally complex encapsulated-ER plasmodesmata. This speculation emerges in part from a consideration of the limitations imposed by passive diffusion on the transport of metabolites and by the necessity of bypassing these limits as multicellularity resulted in larger and larger life-forms. Specifically, manipulation of Fick’s second law of passive diffusion shows that the time it takes for the concentration of a non-electrolyte *j* initially absent from a cell’s interior to reach one-half the concentration of *j* in the external ambient medium (denoted as *t*_0.5_ – *t*_0_) is given by the formula

$$t_{0.5}-t_{0}=\frac{V}{{AP}_{j}}In\frac{(c_{0}-c_{j})t_{0}}{(c_{0}-c_{j})t_{0.5}}=0693\frac{V}{{AP}_{j}},$$

where *V* and *A* are the volume and the surface area of the cell, respectively, *P_j_* is the permeability coefficient of *j* (a constant for any particular non-electrolyte), the expression (*c_o_* – *c_j_*)*t*_0_ is the initial difference between the external and internal concentrations of *j* at time zero, and the expression (*c_o_* – *c_j_*)*t*_0.5_ is the difference between the external and internal concentrations when the internal concentration of *j* reaches one-half that of the ambient medium (Niklas and Spatz, 2012). This formula shows that the time required for passive diffusion to provide essential metabolites to a cell increases in direct proportion to the volume of a cell. Beyond a certain surface area-to-volume limit, passive diffusion must be replaced by bulk flow, which is impossible within a unicellular non-aquatic organism. Consequently, the evolution of complex multicellularity requires intercellular bulk flow that necessitates some form of intercellular “porosity,” e.g., phloem sieve plates. Likewise, intercellular transport systems require cell-type specialization, which has been shown to be positively correlated with genotypic and proteomic “complexity” (e.g., Niklas et al., 2014, 2018; Yruela et al., 2017).

Molecules that regulate plasmodesmata aperture and structure may have performed different functions in the ancestors of land plants. As previously noted, callose turnover is the main contributor to the regulating of plasmodesmata permeability. Although callose is widespread in the plant kingdom, its turnover regulated by plasmodesmata aperture has only been observed in the land plants (Scherp et al., 2001; Schuette et al., 2009). Thus, understanding the functionalities of plasmodesmata-localized proteins implicated in callose turnover could help elucidate the evolution of plasmodesmata structure and plasmodesmata-dependent transport. For example, glycosyl hydrolase 17 (GHL17) belongs to another family of proteins involved in callose degradation (Gaudioso-Pedraza and Benitez-Alfonso, 2014). A phylogenetic study using the sequences of GHL17 of fungi, algae, bryophytes, *Arabidopsis*, and monocots identifies a land plant specific clade characterized by plasmodesmata GHL17 localization (Gaudioso-Pedraza and Benitez-Alfonso, 2014). In contrast, the fungal and algal selected sequences appear to have diverged earlier than the land plant sequences, suggesting a more ancestral GHL17 origin (Gaudioso-Pedraza and Benitez-Alfonso, 2014). Other callose regulation proteins, such as the callose synthase (CalS) family, have been duplicated during the diversification of land plants (Drábková and Honys, 2017). Together, these findings indicate that plasmodesmata-localized proteins were already present in the land plant ancestor but that they played different roles.

As a consequence of plasmodesmata transport, plants can utilize the biogeneric properties of DPMs such as diffusion and lateral inhibition to specify cell identity and develop vascular tissues specialized in transporting nutrients over long distances. Without this capacity, plants would have not been able to generate the complex multicellular organisms that we know and that have become the major life form on earth. Despite the importance of plasmodesmata-mediated transport for plant development and diversification, little is known about their evolution. The reasons for this stem in part from the fact that plasmodesmata have structural characteristics that differ among different kinds of tissues as well as among the different plant lineages, and from the fact that the complete disruption of plant tissues is still challenging (Faulkner and Maule, 2011; Brunkard and Zambryski, 2017). However, some molecules that may be generically involved in the formation of plasmodesmata are now being postulated, as for example certain reticulons (Brunkard and Zambryski, 2017). It is likely that the advent of new methodologies that allow us to identify new plasmodesmata proteins will help elucidate the regulatory properties of plasmodesmata as well as the origins of these molecules and the genes encoding them in organisms that lack or that have less complex plasmodesmata.

## Molecular Regulatory Networks (MRNs): Co-Option, Drift and Plant Evolutionary Transitions

The notion of GRNs, recently referred to as molecular regulatory networks to include other types of molecules, has allowed the fruitful exploration of the collective effect of genes and gene products in organismal development, although several other phenomena have been recently identified that call for a re-evaluation or update of current network modeling formalisms (e.g., the role of intrinsically disordered proteins in gene regulation, Niklas et al., 2015, 2018). Molecular regulatory networks integrate a set of nodes that can stand for genes, proteins, different types of RNA or other molecules, and a set of edges that correspond to the regulatory interactions among the elements represented by nodes. Multiple studies have aimed to study the dynamics of such networks, not only in plants, but also in animals, fungi and bacteria, mostly to test the idea that the steady states (attractors) of molecular regulatory networks correspond to specific cell types or cellular states (Kauffman, 1969; Thomas, 1991; Albert and Othmer, 2003; Alvarez-Buylla et al., 2007)

The picture emerging from theoretical and empirical studies is that molecular regulatory network steady states may indeed correspond to cell types or metabolic states, and that such different attractors can be present even in unicellular organisms that alternate between different phases or states in their life cycle (Quiñones-Valles et al., 2014; Mora Van Cauwelaert et al., 2015). However, the temporal coexistence of different cell types can only occur in multicellular organisms. Multiple studies have suggested that the molecular regulatory networks that underlie the specification of different cell-types in extant multicellular organisms may have been co-opted from multistable molecular regulatory networks, i.e., networks leading to more than one steady state, that were already present in their unicellular ancestors (Newman and Bhat, 2009; Mora Van Cauwelaert et al., 2015; Sebé-Pedrós et al., 2017). Indeed, mathematical and computational models have been used to perform proof-of-principle simulations that illustrate how single cells with multistable molecular regulatory networks can aggregate and couple via diverse communication mechanisms, giving rise to stereotypic and robust arrangements of cells with different identities (Furusawa and Kaneko, 2002; Mora Van Cauwelaert et al., 2015). This is a powerful idea, since this scenario requires no massive or abrupt genetic changes to explain one of the most major evolutionary transitions (Newman and Bhat, 2008, 2009; Niklas and Newman, 2013).

We have argued that some of the basic features of animal and plant body plans may have been generated by the cooption and differential spatiotemporal combination of DPMs (Newman and Bhat, 2008, 2009; Hernández-Hernández et al., 2012; Niklas and Newman, 2013; Niklas, 2014). However, DPMs are associated with molecules that are part of evolving regulatory networks such that DPM-related molecules and their regulatory interactions can change. As these networks evolve, DPMs may become canalized (*sensu* Waddington), this is, the patterns and shapes that were initially generated by generic physico-chemical processes mobilized by a few molecules can become somehow stabilized by the evolution of continuously more robust and intricate regulatory networks (Salazar-Ciudad et al., 2001). Molecular network evolution may also follow a trajectory characterized by developmental system drift (DSD) (True and Haag, 2001), which suggests that genetic networks associated with phenotypes are both flexible and robust, and that differences between regulatory networks in related species arise by elimination or recruitment of new elements, erasing in this way traceable signals of common ancestry at the genetic level. DSD thus suggests that development of homologous traits in related species may not be mediated by homologous genetic factors (Müller and Newman, 1999; Rokas, 2006; Tsong et al., 2006; Kiontke et al., 2007; Nahmad and Lander, 2011; Sommer, 2012; Shbailat and Abouheif, 2013; Stolfi et al., 2014; Arias Del Angel et al., 2017). The mechanisms triggering divergence in the regulatory networks in DSD can involve both *cis*- and *trans*-regulatory changes, and the degree of change can vary from one system to another (Sommer, 2012; Stolfi et al., 2014).

We will now return to the DIF DPM example to illustrate some of these ideas.

All multicellular lineages with cellulosic cell walls appear to have evolved structures analogous to plasmodesmata. Indeed, as noted plasmodesmata evolved independently in different eukaryotic photosynthetic lineages and the molecules associated to their evolutionary origin are still unclear (Brunkard and Zambryski, 2017). However, some of the molecules that passively move through plasmodesmata and that are involved in the DIF DPM may have been co-opted from widely conserved molecular regulatory networks, some of which may predate plant multicellularity.

The case of auxin was briefly mentioned above. Indeed, currently available evidence shows that auxin biosynthesis was already present in the unicellular ancestors of multicellular eukaryotes (Beilby, 2016; Khasin et al., 2017; Ishizaki, 2017; Kato et al., 2017; among many other lines of evidence). However, this is not the case for the auxin transporters that have been identified and thoroughly studied in angiosperm model systems. It has thus been suggested that auxin initially moved only through plasmodesmata in a passive manner, contributing to multicellular organization through the formation of gradients and concentration patterns that could account for differential cellular behaviors and identities in vascular land plants, some algae, and bryophytes. If true, auxin transport seems to have been canalized and greatly potentialized by the evolution of complex molecular networks associated with its biosynthesis and transport. So much so, that in plants like *Arabidopsis* auxin local concentration is highly regulated and participates in diverse developmental processes and events under specific spatiotemporal conditions. Moreover, such tight regulation of auxin transport has enabled cellular and organ polarization, and has likely contributed to other evolutionary transitions, such as that to vascular plants. It also seems to be the case that extant networks associated with auxin biosynthesis and transport differ in particular elements and interactions, suggesting that the mechanisms of canalization have differed among plants or that some degree of developmental systems drift has occurred.

With regard to the DIF DPM, the role of molecular regulatory network cooption and further canalization is illustrated by the MYB-bHLH-WD40 protein complex. These plant proteins are involved in complex networks that act in different plant organs and developmental stages, enabling the determination of diverse cell types, such as stomata, pavement cells, trichomes, and trichoblasts (Ramsay and Glover, 2005; Benítez et al., 2011; Torii, 2012; Horst et al., 2015; Breuninger et al., 2016). A central feature of this complex is that some of its components may move to neighboring cells through plasmodesmata, which gives rise to the coupling of otherwise intracellular networks and, concomitantly, the emergence of stereotypic cellular arrangements. Indeed, it is by the intercellular transport and mutual regulation of MYB, bHLH, and WD40 proteins that some of the well-known patterns of spaced-out stomata, trichomes, and aligned root hairs arise during plant development (Benítez et al., 2011; Torii, 2012; Horst et al., 2015). Interestingly, although the molecular regulatory networks in which these proteins take part seem to have come together in land plants, their key components appear to predate plant multicellularity (Ramsay and Glover, 2005). Consequently, some of the major events in the diversification of cellular types and functions have involved the co-option of ancient molecules, the presence of plasmodesmata, and the associated mobilization of certain DPMs, even if the MYB-bHLH-WD40 complex has drifted into regulatory systems that are currently species- or even organ-dependent.

## Final Remarks

**FIGURE 7:**
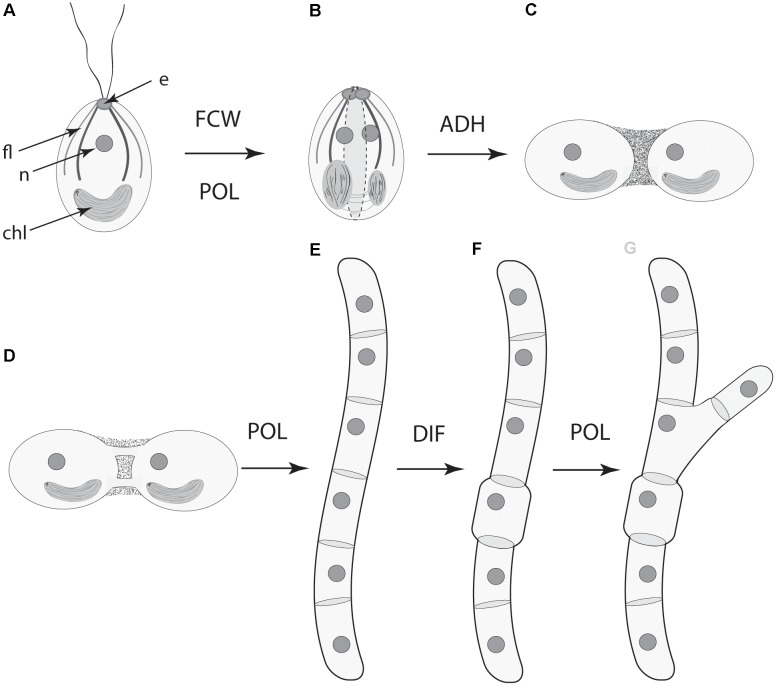
Serial schematic of the ways four DPMs have contributed to the evolution of complex multicellularity in the land plants (see also, **Figure 5**). **(A)** The ancestral unicellular condition within the Streptophytes (the green algae and the land plants) with a flagellar apparatus and associated eyespot, nucleus, and parietal chloroplast. **(B)** The future cell wall (FCM) module operates along the longitudinal cell axis in conjunction with the polarity (POL) module to specify the plane of cell division (dashed lines). **(C)** The cell-to-cell adhesion (ADH) module establishes the colonial body plan (show here by two adjoining cells). **(D)** Symplastic continuity between adjoining cells is established by means of plasmodesmata. **(E)** The POL module shifts the frame of reference for the FCW module (now orthogonal to the cell longitudinal axis) to form an unbranched filament. **(F)** The differentiation (DIF) module is deployed for the evolution of specialized cells. **(G)** The POL module operates to now specify a second plain of cell division to produce a branched filament. The role of the POL module in establishing a third plane of cell division (and thus the development of parenchyma) is not shown here. e, eyespot; fl., flagellar apparatus; n, nucleus; chl, chloroplast.

Based on our review of the available evidence, we reach the following conclusions:

• Dynamical patterning modules, defined as sets of conserved gene products and molecular networks in conjunction with the physical morphogenetic and patterning processes they mobilize, have played ubiquitous and central roles in the evolution of multicellularity in the algae, land plants, fungi, as they have been shown to in metazoans (schematized in **Figure 7**).

• Four DPMs are critical in the context of multicellular evolution of plants and fungi: the DPM for the orientation of the future cell wall (FCW), the DPM for cell-to-cell adhesion (ADH), the DPM for polarity (POL), and the DPM for differentiation (DIF).
• Comparisons across the various fungal, algal, and land plant lineages indicate that these four DPMs have recruited different mechanisms and materials to achieve the same ends. For example, the materials used to achieve cell-to-cell adhesion differ dramatically among the various multicellular algal lineages and the land plants.
• These differences in the materials recruited by the various DPMs indicate that natural selection operates on morphological phenotypes and not on the components and mechanisms that produce them.

As exemplified in this study focusing on plant multicellularity, the DPM concept provides a valuable framework to further understand the processes behind multicellular development and evolution and can give rise to clear propositions that can in turn be tested through comparative methods, mathematical and computational modeling, and experimental modification of parameters and biogeneric properties. However, the DPM concept has not been fully integrated into the “standard model” of contemporary evolutionary developmental biology. Typically, “mechanism” is considered at the level of genes and gene networks, while morphology is handled descriptively, with adaptationist narratives where they pertain, and appeals to pleiotropy and its consequences when they do not (Minelli, 2018). This perspective is unsatisfactory as an explanatory framework for biological form in light of the unquestioned role of physical mechanisms of morphogenesis across all categories of multicellular (Forgacs and Newman, 2005; Niklas and Newman, 2013; Newman and Niklas, 2018) and increased recognition of the conservation of early-evolved architectural motifs despite drift in molecular mechanisms (True and Haag, 2001).

Such homoplasy is even more pervasive in plant than in animal systems, where, as we have described here and elsewhere, there have been multiple routes to multicellularity rather than the single, classical cadherin-based, one in the metazoans (Niklas and Newman, 2013; Newman, 2016b). Moreover, the ability of the cyanobacteria, the land plants, and the brown algae to form plasmodesmata-like intercellular structures involves significantly different GRNs, gene products, and developmental processes. Yet, the result in each case is the same, i.e., intercellular adhesion, communication, and polarity.

Whereas in animal systems GRNs and DPMs act relatively independently of each other, with the former mainly specifying cell type identity and latter patterns and arrangements of cells (Newman et al., 2009), the molecular regulatory networks of plants and fungi act in a more integrated fashion, comprising both GRN- and DPM-type functions of metazoans. This is partly because transcription factors move more freely between cells in the non-metazoans. Moreover, since the physics embodied in DPMs often leads to predicable morphological outcomes, these modules have served as “simplification forces” in evolution, acting as major instructive cues that channel development in both animals and plants. In contrast to animal GRNs, however, the mixed-nature plant molecular regulatory networks have been “complexification forces” in plant and fungal evolution, offering additional opportunities to use/modulate/bridge DPMs to generate an enhanced spectrum of morphological complexity.

The behaviors of developing tissues as excitable biogeneric materials (liquids and liquid crystals in the case of animals, deformable cellular solids in the case of plants), are inescapable, as are the preferred morphological motifs generated by characteristic DPMs of these materials, whatever their molecular genetic underpinnings may be. Understanding these inherent properties is essential to mechanistic explanations of development and its transformations during the evolution of multicellular organisms (Newman, 2017). A challenge for future research is to determine how these modules recruit and integrate the ancillary processes required to achieve the morphological variety seen across the broad phylogenetic spectrum of multicellular plants and fungi.

## Author Contributions

All authors listed have made a substantial, direct and intellectual contribution to the work, and approved it for publication.

## Conflict of Interest Statement

The authors declare that the research was conducted in the absence of any commercial or financial relationships that could be construed as a potential conflict of interest.

## References

[B1] Alassimone J. Naseer S. Geldner N. (2010). A developmental framework for endodermal differentiation and polarity. *Proc. Natl. Acad. Sci. U.S.A.* 107 5214–5219. 10.1073/pnas.0910772107 20142472 PMC2841941

[B2] Albert R. Othmer H. G. (2003). The topology of the regulatory interactions predicts the expression pattern of the segment polarity genes in *Drosophila melanogaster*. *J. Theor. Biol.* 223 1–18. 10.1016/S0022-5193(03)00035-3 12782112 PMC6388622

[B3] Alvarez-Buylla E. R. Benítez M. Dávila E. B. Chaos A. Espinosa-Soto C. Padilla-Longoria P. (2007). Gene regulatory network models for plant development. *Curr. Opin. Plant Biol.* 10 83–91. 10.1016/j.pbi.2006.11.008 17142086

[B4] Arias Del Angel J. A. Escalante A. E. Martinez-Castilla L. P. Benitez M. (2017). An evo-devo perspective on multicellular development of Myxobacteria. *J. Exp. Zool. B Mol. Dev. Evol.* 328 165–178. 10.1002/jez.b.22727 28217903

[B5] Beaumont N. (2009). Modelling the transport of nutrients in early animals. *Evol. Biol.* 36 256–266. 10.1007/s11692-008-9047-2

[B6] Beilby M. J. (2016). Multi-scale characean experimental system: from electrophysiology of membrane transporters to cell-to-cell connectivity, cytoplasmic streaming and auxin metabolism. *Front. Plant Sci.* 7:1052. 10.3389/fpls.2016.01052 27504112 PMC4958633

[B7] Benítez M. Hejatko J. (2013). Dynamics of cell-fate determination and patterning in the vascular bundles of *Arabidopsis thaliana*. *PLoS One* 8:e63108. 10.1371/journal.pone.0063108 23723973 PMC3664626

[B8] Benítez M. Monk N. A. Alvarez-Buylla E. R. (2011). Epidermal patterning in *Arabidopsis*: models make a difference. *J. Exp. Zool. B Mol. Dev. Evol.* 316 241–253. 10.1002/jez.b.21398 21259417

[B9] Benitez-Alfonso Y. Faulkner C. Pendle A. Miyashima S. Helariutta Y. Maule A. (2013). Symplastic intercellular connectivity regulates lateral root patterning. *Dev. Cell* 26 136–147. 10.1016/j.devcel.2013.06.010 23850190

[B10] Bloemendal S. Kück U. (2013). Cell-to-cell communication in plants, animals, and fungi: a comparative review. *Naturwissenschaften* 100 3–19. 10.1007/s00114-012-0988 23128987

[B11] Bonner J. T. (2000). *First Signals: The Evolution of Multicellular Development.* Princeton, NJ: Princeton University Press.

[B12] Breuninger H. Thamm A. Streubel S. Sakayama H. Nishiyama T. Dolan L. (2016). Diversification of a transcription factor family led to the evolution of antagonistically acting genetic regulators of root hair growth. *Curr. Biol.* 26 1622–1628. 10.1016/j.cub.2016.04.060 27265398 PMC4920954

[B13] Brunkard J. O. Zambryski P. C. (2017). Plasmodesmata enable multicellularity: new insights into their evolution, biogenesis, and functions in development and immunity. *Curr. Opin. Plant Biol.* 35 76–83. 10.1016/j.pbi.2016.11.007 27889635

[B14] Cannon M. C. Terneus K. Hall Q. Tan L. Wang Y. Wegenhart B. L. (2008). Self-assembly of the plant cell wall requires an extension scaffold. *Proc. Natl. Acad. Sci. U.S.A.* 105 2226–2231. 10.1073/pnas.0711980105 18256186 PMC2538902

[B15] Carlsbecker A. Lee J. Y. Roberts C. J. Dettmer J. Lehesranta S. Zhou J. (2010). Cell signalling by microRNA165/6 directs gene dose-dependent root cell fate. *Nature* 465 316–321. 10.1038/nature08977 20410882 PMC2967782

[B16] Christensen N. M. Faulkner C. Oparka K. (2009). Evidence for unidirectional flow through plasmodesmata. *Plant Physiol.* 150 96–104. 10.1104/pp.109.137083 19270059 PMC2675744

[B17] Cook M. Graham L. Botha C. Lavin C. (1997). Comparative ultrastructure of plasmodesmata of Chara and selected bryophytes: toward an elucidation of the evolutionary origin of plant plasmodesmata. *Am. J. Bot.* 84:1169. 10.2307/2446040 21708671

[B18] Cook M. Graham L. Van Bel A. J. E. van Kesteren W. J. P. (1999). “Evolution of plasmodesmata,” in *Plasmodesmata: Structure, Function, Role in Cell Communication*, eds van Bel A. J. E. Kesteren W. J. P. (Berlin: Springer).

[B19] Cui H. Levesque M. P. Vernoux T. Jung J. W. Paquette A. J. Gallagher K. L. (2007). An evolutionarily conserved mechanism delimiting SHR movement defines a single layer of endodermis in plants. *Science* 316 421–425. 10.1126/science.1139531 17446396

[B20] De Storme N. Geelen D. (2014). Callose homeostasis at plasmodesmata: molecular regulators and developmental relevance. *Front. Plant Sci.* 5:138. 10.3389/fpls.2014.00138 24795733 PMC4001042

[B21] Dettmer J. Friml J. (2011). Cell polarity in plants: when two do the same, it is not the same. *Curr. Opin. Cell Biol.* 23 686–696. 10.1016/j.ceb.2011.09.006 21962973

[B22] Drábková L. Z. Honys D. (2017). Evolutionary history of callose synthases in terrestrial plants with emphasis on proteins involved in male gametophyte development. *PLoS One* 12:e0187331. 10.1371/journal.pone.0187331 29131847 PMC5683620

[B23] Faulkner C. Maule A. (2011). Opportunities and successes in the search for plasmodesmal proteins. *Protoplasma* 248 27–38. 10.1007/s00709-010-0213-x 20922549

[B24] Ferris P. J. Woessner J. P. Waffenschmidt S. Kilz S. Drees J. Goodenough U. W. (2001). Glycosylated polyproline II rods with kinks as a structural motif in plant hydroxyproline-rich glycoproteins. *Biochemistry* 40 2978–2987. 10.1021/bi0023605 11258910

[B25] Fleury V. (1999). Un possible lien entre la croissance dendritique en physique et la morphogenèse des plantes. *C. R. Acad. Sci. Paris Ser. III Sci. Vie* 322 725–734. 10.1016/S0764-4469(00)80030-X

[B26] Floyd G. L. Stewart K. D. Mattox K. R. (197l). Cytokinesis and plasmodesmata in Ulothrix. *J. Phycol.* 7 306–309.

[B27] Folse H. J. Jr. Roughgarden J. (2010). What is an individual organism? A multilevel selection perspective. *Q. Rev. Biol.* 85 447–472. 10.1086/656905 21243964

[B28] Forgacs G. Newman S. A. (2005). *Biological Physics of the Developing Embryo.* Cambridge: Cambridge University Press 10.1017/CBO9780511755576

[B29] Fraser T. W. Gunning B. E. S. (1969). The ultrastructure of plasmodesmata in the filamentous green alga, *Bulbochaete hiloensis* (Nordst.) tiffany. *Planta* 88 244–254. 10.1007/BF00385067 24504895

[B30] Furusawa C. Kaneko K. (2002). Origin of multicellular organisms as an inevitable consequence of dynamical systems. *Anat. Rec.* 268 327–342. 10.1002/ar.10164 12382328

[B31] Galletti R. Verger S. Hamant O. Ingram G. C. (2016). Developing a ‘thick skin’: a paradoxical role for mechanical tension in maintaining epidermal integrity? *Development* 143 3249–3258. 10.1242/dev.132837 27624830

[B32] Gardiner J. (2013). The evolution and diversification of plant microtubule-associated proteins. *Plant J.* 75 219–229. 10.1111/tpj.12189 23551562

[B33] Garrett J. J. Meets M. J. Blackshaw M. T. Blackshaw L. C. Hou H. Styranko D. M. (2012). A novel, semi-dominant allele of MONOPTEROS provide insight into leaf initiation and vein pattern formation. *Planta* 236 297–312. 10.1007/s00425-012-1607-0 22349732

[B34] Gaudioso-Pedraza R. Benitez-Alfonso Y. (2014). A phylogenetic approach to study the origin and evolution of plasmodesmata-localized glycosyl hydrolases family 17. *Front. Plant Sci.* 5:212. 10.3389/fpls.2014.00212 24904609 PMC4033164

[B35] Geldner N. (2009). Cell polarity in plants—A PARspective on PINs. *Curr. Opin. Plant Biol.* 12 42–48. 10.1016/j.pbi.2008.09.009 18993110

[B36] Graham L. E. Graham J. M. Wilcox L. W. (2009). *Algae*, 2nd Edn San Francisco, CA: Benjamin Cummings.

[B37] Green K. J. Viamontes G. I. Kirk D. L. (1981). Mechanism of formation, ultrastructure and function of the cytoplasmic bridge system during morphogenesis in *Volvox*. *J. Cell Biol.* 91 756–769. 10.1083/jcb.91.3.756 7328120 PMC2112823

[B38] Green S. Batterman R. (2017). Biology meets physics: reductionism and multi-scale modeling of morphogenesis. *Stud. Hist. Philos. Biol. Biomed. Sci.* 61 20–34. 10.1016/j.shpsc.2016.12.003 28024174

[B39] Grosberg R. K. Strathmann R. (2007). The evolution of multicellularity: a minor major transition? *Annu. Rev. Ecol. Evol. Syst.* 38 621–654. 10.1146/annurev.ecolsys.36.102403.114735

[B40] Guenoune-Gelbart D. Elbaum M. Sagi G. Levy A. Epel B. L. (2008). *Tobacco mosaic virus* (TMV) replicase and movement protein function synergistically in facilitating TMV spread by lateral diffusion in the plasmodesmal desmotubule of *Nicotiana benthamiana*. *Mol. Plant Microbe Interact.* 21 335–345. 10.1094/MPMI-21-3-0335 18257683

[B41] Guseman J. M. Lee J. S. Bogenschutz N. L. Peterson K. M. Virata R. E. Xie B. (2010). Dysregulation of cell-to-cell connectivity and stomatal patterning by loss-of-function mutation in *Arabidopsis CHORUS* (*GLUCAN SYNTHASE-LIKE 8*). *Development* 137 1731–1741. 10.1242/dev.049197 20430748

[B42] Hall J. D. McCourt R. M. Delwiche C. F. (2008). Patterns of cell division in the filamentous Desmidiaceae, close green algal relatives of land plants. *Am. J. Bot.* 95 643–654. 10.3732/ajb.2007210 21632389

[B43] Han X. Hyun T. K. Zhang M. Kumar R. Koh E. J. Kang B. H. (2014). Auxin-callose-mediated plasmodesmal gating is essential for tropic auxin gradient formation and signaling. *Dev. Cell* 28 132–146. 10.1016/j.devcel.2013.12.008 24480642

[B44] Hernández-Hernández V. Niklas K. J. Newman S. A. Benítez M. (2012). Dynamical patterning modules in plant development and evolution. *Int. J. Dev. Biol.* 56 661–674. 10.1387/ijdb.120027mb 23319343

[B45] Herron M. D. Michod R. E. (2008). Evolution of complexity in the volvocine algae: transitions in individuality through Darwin’s eye. *Evolution* 62 436–451. 10.1111/j.1558-5646.2007.00304.x 18031303

[B46] Hoops H. J. Nishii I. Kirk D. L. (2000). “Cytoplasmic bridges in *Volvox* and its relatives,” in *Madame Curie Bioscience Database [Internet]* (Austin, TX: Landes Bioscience). Available at: http://www.ncbi.nlm.nih.gov/books/NBK6424/

[B47] Horst R. J. Fujita H. Lee J. S. Rychel A. L. Garrick J. M. Kawaguchi M. (2015). Molecular framework of a regulatory circuit initiating two-dimensional spatial patterning of stomatal lineage. *PLoS Genet.* 11:e1005374. 10.1371/journal.pgen.1005374 26203655 PMC4512730

[B48] Ishizaki K. (2017). Evolution of land plants: insights from molecular studies on basal lineages. *Biosci. Biotechnol. Biochem.* 81 73–80. 10.1080/09168451.2016.1224641 27595342

[B49] Kato H. Nishihama R. Weijers D. Kohchi T. (2017). Evolution of nuclear auxin signaling: lessons from genetic studies with basal land plants. *J. Exp. Bot.* 69 291–301. 10.1093/jxb/erx267 28992186

[B50] Kauffman S. A. (1969). Metabolic stability and epigenesis in randomly constructed genetic nets. *J. Theor. Biol.* 22 437–467. 10.1016/0022-5193(69)90015-0 5803332

[B51] Khasin M. Cahoon R. R. Nickerson K. W. Riekhof W. R. (2017). Molecular machinery of auxin synthesis, secretion, and perception in the unicellular chlorophyte alga *Chlorella sorokiniana* UTEX 1230. *bioRxiv* 10.1101/172833 [Preprint]. PMC6287815 30532131

[B52] Kiontke K. Barriere A. Kolotuev I. Podbilewicz B. Sommer R. Fitch D. H. (2007). Trends, stasis, and drift in the evolution of nematode vulva development. *Curr. Biol.* 17 1925–1937. 10.1016/j.cub.2007.10.061 18024125

[B53] Kirk D. L. (2005). A twelve-step program for evolving multicellularity and a division of labor. *Bioessays* 27 299–310. 10.1002/bies.20197 15714559

[B54] Kitagawa M. Fujita T. (2013). Quantitative imaging of directional transport through plasmodesmata in moss protonemata via single-cell photoconversion of Dendra2. *J. Plant Res.* 126 577–585. 10.1007/s10265-013-0547-5 23381037 PMC4194024

[B55] Knoll A. (2011). The multiple origins of complex multicellularity. *Annu. Rev. Earth Planet. Sci.* 39 217–239. 10.1146/annurev.earth.031208.100209

[B56] Kutschera U. Niklas K. J. (2005). Endosymbiosis, cell evolution, and speciation. *Theory Biosci.* 124 1–24. 10.1016/j.thbio.2005.04.001 17046345

[B57] Kutschera U. Niklas K. J. (2007). The epidermal-growth-control theory of stem elongation: an old and new perspective. *Plant Physiol.* 164 1395–1409. 10.1016/j.jplph.2007.08.002 17905474

[B58] Kutschera U. Niklas K. J. (2008). Macroevolution via secondary endosymbiosis: a Neo- Goldschmidtian view of unicellular hopeful monsters and Darwin’s primordial intermediate form. *Theory Biosci.* 127 277–289. 10.1007/s12064-008-0046-8 18581157

[B59] Kwiatkowska M. (1999). “Plasmodesmal coupling and cell differentiation in algae,” in *Plasmodesmata: Structure, Function, Role in Cell Communication*, eds Van Bel A. J. E. van Kesteren W. J. P. (Berlin: Springer).

[B60] Lamport D. T. A. Kieliszewski M. J. Chen Y. Cannon M. C. (2011). Role of the extension superfamily in primary cell wall architecture. *Plant Physiol.* 156 11–19. 10.1104/pp.110.169011 21415277 PMC3091064

[B61] Levine H. Ben-Jacob E. (2004). Physical schemata underlying biological pattern formation-examples, issues and strategies. *Phys. Biol.* 1 P14–P22. 10.1088/1478-3967/1/2/P01 16204813

[B62] Lucas W. I. Ding B. Van der Schoot C. (1993). Plasmodesmata and the supracellular nature of plants. *New Phytol.* 125 435–476. 10.1111/j.1469-8137.1993.tb03897.x 33874589

[B63] Lund E. J. (1947). *Bioelectric Fields and Growth.* Austin, TX: University of Texas Press.

[B64] Majda M. Grones P. Sinton I.-M. Vain T. Milani P. Krupinski P. (2017). Mechanochemical polarization of contiguous cell walls shapes plant pavement cells. *Dev. Cell* 43 290–304. 10.1016/j.devcel.2017.10.017 29112850

[B65] Marchant H. J. (1976). “Plasmodesmata in algae and fungi,” in *Intercellular Communication in Plants: Studies on Plasmodesmata*, eds Gunning B. E. S. Robards A. W. (New York, NY: Springer), 59–78.

[B66] McGhee G. R. (1999). *Theoretical Morphology: The Concept and Its Applications.* New York, NY: Columbia University Press.

[B67] Menzel D. (1996). The role of the cytoskeleton in polarity and morphogenesis of algal cells. *Curr. Opin. Cell Biol.* 1 38–42. 10.1016/S0955-0674(96)80046-9 8791401

[B68] Meyerowitz E. M. (2002). Plants compared to animals: the broadest comparative study of development. *Science* 295 1482–1485. 10.1126/science.1066609 11859185

[B69] Minelli A. (2018). *Plant Evolutionary Developmental Biology: The Evolvability of the Phenotype.* Cambridge: Cambridge University Press 10.1017/9781139542364

[B70] Mora Van Cauwelaert E. Del Angel A. Antonio J. Benítez M. Azpeitia E. M. (2015). Development of cell differentiation in the transition to multicellularity: a dynamical modeling approach. *Front. Microbiol.* 6:603. 10.3389/fmicb.2015.00603 26157427 PMC4477168

[B71] Müller G. B. Newman S. A. (1999). “Generation, integration, autonomy: three steps in the evolution in homology,” in *Homology* Vol. 222 eds Bock G. K. Cardew G. (Chichester: Wiley), 65–73. 10.1002/9780470515655.ch5 10332753

[B72] Nahmad M. Lander A. D. (2011). Spatiotemporal mechanisms of morphogen gradient interpretation. *Curr. Opin. Genet. Dev.* 21 726–731. 10.1016/j.gde.2011.10.002 22033220 PMC3423890

[B73] Newman S. A. (2011). The developmental specificity of physical mechanisms. *Ludus Vitalis* 19 343–351.

[B74] Newman S. A. (2012). Physico-genetic determinants in the evolution of development. *Science* 338 217–219. 10.1126/science.1222003 23066074

[B75] Newman S. A. (2016a). ‘Biogeneric’ developmental processes: drivers of major transitions in animal evolution. *Philos. Trans. R. Soc. Lond. B Biol. Sci.* 371:20150443. 10.1098/rstb.2015.0443 27431521 PMC4958937

[B76] Newman S. A. (2016b). “Multicellularity, the emergence of animal body plans, and the stabilizing role of the egg,” in *Multicellularity: Origins and Evolution*, eds Niklas K. J. Newman S. A. (Cambridge, MA: MIT Press), 225–246.

[B77] Newman S. A. (2017). “Inherency,” in *Evolutionary Developmental Biology*, eds Nuño de la Rosa L. Müller G. B. (Basel: Springer). 10.1007/978-3-319-33038-9_78-1

[B78] Newman S. A. Bhat R. (2008). Dynamical patterning modules: physico-genetic determinants of morphological development and evolution. *Phys. Biol.* 5:15008. 10.1088/1478-3975/5/1/015008 18403826

[B79] Newman S. A. Bhat R. (2009). Dynamical patterning modules: a “pattern language” for development and evolution of multicellular form. *Int. J. Dev. Biol.* 53 693–705. 10.1387/ijdb.072481sn 19378259

[B80] Newman S. A. Bhat R. Mezentseva N. V. (2009). Cell state switching factors and dynamical patterning modules: complementary mediators of plasticity in development and evolution. *J. Biosci.* 34 553–572. 10.1007/s12038-009-0074-7 19920341

[B81] Newman S. A. Niklas K. J. (2018). “Dynamical patterning modules link genotypes to morphological phenotypes in multicellular evolution,” in *Cells in Evolutionary Biology*, eds Hall B. K. Moody S. A. (Boca Raton, FL: CRC Press), 235–266.

[B82] Niklas K. J. (1989). Mechanical behavior of plant tissues as inferred from the theory of pressurized cellular solids. *Am. J. Bot.* 76 929–937. 10.1002/j.1537-2197.1989.tb15071.x

[B83] Niklas K. J. (1992). *Plant Biomechanics.* Chicago, IL: University of Chicago Press.

[B84] Niklas K. J. (1994). *Plant Allometry.* Chicago, IL: University of Chicago Press.

[B85] Niklas K. J. (2000). The evolution of plant body plans —- A biomechanical perspective. *Ann. Bot.* 85 411–438. 10.1006/anbo.1999.1100

[B86] Niklas K. J. (2014). The evolutionary-developmental origins of multicellularity. *Am. J. Bot.* 101 6–25. 10.3732/ajb.1300314 24363320

[B87] Niklas K. J. (2017). Size-dependent variation in plant form. *Curr. Biol.* 27 R853–R909. 10.1016/j.cub.2017.02.007 28898662

[B88] Niklas K. J. Bondos S. E. Dunker A. K. Newman S. A. (2015). Rethinking gene regulatory network theory in light of alternative splicing, intrinsically disordered protein domains, and post-translational modifications. *Front. Cell Dev. Biol.* 3:8 10.3389/fcell.2015.00008 PMC4341551 25767796

[B89] Niklas K. J. Cobb E. D. Crawford D. R. (2013). The evo-devo of multicellular cells, tissues and organisms, and an alternative route to multicellularity. *Evol. Dev.* 15 466–474. 10.1111/ede.12055 24261447

[B90] Niklas K. J. Cobb E. D. Dunker A. K. (2014). The number of cell types, information content, and the evolution of multicellularity. *Acta Soc. Bot. Pol.* 83 337–347. 10.5586/asbp.2014.034

[B91] Niklas K. J. Dunker A. K. Yruela I. (2018). The evolutionary origins of cell type diversification and the role of intrinsically disordered proteins. *J. Exp. Bot.* 69 1427–1446. 10.1093/jxb/erx493 29394379

[B92] Niklas K. J. Kaplan D. R. (1991). “Biomechanics and the adaptive significance of multicellularity in plants,” in *Proceedings of the Fourth International Congress of Systematics and Evolutionary Biology: The Unity of Evolutionary Biology*, ed. Dudley E. C. (Portland, OR: Dioscorides Press), 489–502.

[B93] Niklas K. J. Newman S. A. (2013). The origins of multicellular organisms. *Evol. Dev.* 15 41–52. 10.1111/ede.12013 23331916

[B94] Niklas K. J. Spatz H.-C. (2012). *Plant Physics.* London: University of Chicago Press 10.7208/chicago/9780226586342.001.0001

[B95] Peaucelle A. Wightman R. Höfte H. (2015). The control of growth symmetry breaking in the *Arabidopsis* hypocotyl. *Curr. Biol.* 25 1746–1752. 10.1016/j.cub.2015.05.022 26073136

[B96] Pillitteri L. J. Dong J. (2013). Stomatal development in Arabidopsis. *Arabidopsis Book* 11:e0162. 10.1199/tab.0162 23864836 PMC3711358

[B97] Prochiantz A. (2011). Homeoprotein intercellular transfer, the hidden face of cell—Penetrating peptides. *Methods Mol. Biol.* 683 249–257. 10.1007/978-1-60761-919-2_18 21053135

[B98] Quiñones-Valles C. Sánchez-Osorio I. Martínez-Antonio A. (2014). Dynamical modeling of the cell cycle and cell fate emergence in *Caulobacter crescentus*. *PLoS One* 9:e111116. 10.1371/journal.pone.0111116 25369202 PMC4219702

[B99] Ramsay N. A. Glover B. J. (2005). MYB–bHLH–WD40 protein complex and the evolution of cellular diversity. *Trends Plant Sci.* 10 63–70. 10.1016/j.tplants.2004.12.011 15708343

[B100] Ranjan A. Townsley B. T. Ichihashi Y. Sinha N. R. Chitwood R. H. (2015). An intracellular transcriptomic atlas of the giant coenocyte *Caulerpa taxifolia*. *PLoS Genet.* 11:e1004900. 10.1371/journal.pgen.1004900 25569326 PMC4287348

[B101] Raven J. A. (1997). Miniview: multiple origins of plasmodesmata. *Eur. J. Phycol.* 32 95–101. 10.1080/09670269710001737009

[B102] Rokas A. (2006). Evolution: different paths to the same end. *Nature* 443 401–402. 10.1038/443401a 17006502

[B103] Ruan Y. L. Xu S. M. White R. Furbank R. T. (2004). Genotypic and developmental evidence for the role of plasmodesmatal regulation in cotton fiber elongation mediated by callose turnover. *Plant Physiol.* 136 4104–4113. 10.1104/pp.104.051540 15557097 PMC535841

[B104] Sager R. Lee J. Y. (2014). Plasmodesmata in integrated cell signalling: insights from development and environmental signals and stresses. *J. Exp. Bot.* 65 6337–6358. 10.1093/jxb/eru365 25262225 PMC4303807

[B105] Salazar-Ciudad I. Newman S. A. Solé R. (2001). Phenotypic and dynamical transitions in model genetic networks. I. Emergence of patterns and genotype-phenotype relationships. *Evol. Dev.* 3 84–94. 10.1046/j.1525-142x.2001.003002084.x 11341677

[B106] Schaefer E. Belcram K. Uyttewaal M. Duroc Y. Goussot M. Legland D. (2017). The preprophase band of microtubules controls the robustness of division orientation in plants. *Proc. Natl. Acad. Sci. U.S.A.* 356 186–189. 10.1126/science.aal3016 28408602

[B107] Scherp P. Grotha R. Kutschera U. (2001). Occurrence and phylogenetic significance of cytokinesis-related callose in green algae, bryophytes, ferns and seed plants. *Plant Cell Rep.* 20 143–149. 10.1007/s002990000301 30759901

[B108] Schuette S. Wood A. J. Geisler M. Geisler-Lee J. Ligrone R. Renzaglia K. S. (2009). Novel localization of callose in the spores of *Physcomitrella patens* and phylogenomics of the callose synthase gene family. *Ann. Bot.* 103 749–756. 10.1093/aob/mcn268 19155219 PMC2707875

[B109] Sebé-Pedrós A. Degnan B. M. Ruiz-Trillo I. (2017). The origin of Metazoa: a unicellular perspective. *Nat. Rev. Genet.* 18 498–512. 10.1038/nrg.2017.21 28479598

[B110] Seiler S. Justa-Schuch D. (2010). Conserved components, but distinct mechanisms for the placement and assembly of the cell division machinery in unicellular and filamentous ascomycetes. *Mol. Microbiol.* 78 1058–1076. 10.1111/j.1365-2958.2010.07392.x 21091496

[B111] Shbailat S. J. Abouheif E. (2013). The wing-patterning network in the wingless castes of Myrmicine and Formicine ant species is a mix of evolutionarily labile and non-labile genes. *J. Exp. Zool. B Mol. Dev. Evol.* 320 74–83. 10.1002/jez.b.22482 23225600

[B112] Shin K. Fogg V. C. Margolis B. (2006). Tight junctions and cell polarity. *Annu. Rev. Cell Dev. Biol.* 22 207–235. 10.1146/annurev.cellbio.22.010305.104219 16771626

[B113] Sideman E. J. Scheirer D. C. (1977). Some fine structural observations on developing and mature sieve elements in the brown alga *Laminaria saccharina*. *Am. J. Bot.* 64 649–657. 10.1002/j.1537-2197.1977.tb11905.x

[B114] Sinha S. Sridhar S. (2015). *Patterns in Excitable Media: Genesis, Dynamics, and Control.* Boca Raton, FL: CRC Press.

[B115] Sommer R. J. (2012). Evolution of regulatory networks: nematode vulva induction as an example of developmental systems drift. *Adv. Exp. Med. Biol.* 751 79–91. 10.1007/978-1-4614-3567-9_4 22821454

[B116] Stanley M. S. Perry R. M. Callow J. A. (2005). Analysis of expressed sequence tags (ESTs) from the green alga *Ulva linza* (Chlorophyta). *J. Phycol.* 41 1219–1226. 10.1111/j.1529-8817.2005.00138.x

[B117] Stewart K. D. Mattox K. R. Floyd G. L. (1973). Mitosis, cytokinesis, the distribution of plasmodesmata, and other cytological characteristics in the Ulotrichales, Ulvales, and Chaetophorales: phylogenetic and taxonomic considerations. *J. Phycol.* 9 128–141. 10.1111/j.1529-8817.1973.tb04068.x

[B118] Stolfi A. Lowe E. K. Racioppi C. Ristoratore F. Brown C. T. Swalla B. J. (2014). Divergent mechanisms regulate conserved cardiopharyngeal development and gene expression in distantly related ascidians. *eLife* 3:e03728. 10.7554/eLife.03728 25209999 PMC4356046

[B119] Sumper M. Hallmann A. (1998). Biochemistry of the extracellular matrix of *Volvox*. *Int. Rev. Cytol.* 180 51–85. 10.1016/S0074-7696(08)61770-2 9496634

[B120] Swift J. Ivanovska I. L. Buxboim A. Harada T. Dingal P. C. Pinter J. (2013). Nuclear lamin-A scales with tissue stiffness and enhances matrix- directed differentiation. *Science* 341:1240104. 10.1126/science.1240104 23990565 PMC3976548

[B121] Thomas R. (1991). Regulatory networks seen as asynchronous automata: a logical description. *J. Theor. Biol.* 153 1–23. 10.1016/S0022-5193(05)80350-9

[B122] Torii K. U. (2012). Two-dimensional spatial patterning in developmental systems. *Trends Cell Biol.* 22 438–446. 10.1016/j.tcb.2012.06.002 22789547

[B123] True J. R. Haag E. S. (2001). Developmental system drift and flexibility in evolutionary trajectories. *Evol. Dev.* 3 109–119. 10.1046/j.1525-142x.2001.003002109.x 11341673

[B124] Tsong A. E. Tuch B. B. Li H. Johnson A. D. (2006). Evolution of alternative transcriptional circuits with identical logic. *Nature* 443 415–420. 10.1038/nature05099 17006507

[B125] Tylewicz S. Petterle A. Marttila S. Miskolczi P. Azeez A. Singh R. K. (2018). Photoperiodic control of seasonal growth is mediated by ABA acting on cell-cell communication. *Science* 360 212–214. 10.1126/science.aan8576 29519919

[B126] Urbanus S. L. Martinelli A. P. Dinh Q. D. P. Aizza L. C. B. Dornelas M. C. Angenent G. C. (2010). Intercellular transport of epidermis-expressed MADS domain transcription factors and their effect on plant morphology and floral transition. *Plant J.* 63 60–72. 10.1111/j.1365-313X.2010.04221.x 20374529

[B127] Vatén A. Dettmer J. Wu S. Stierhof Y. D. Miyashima S. Yadav S. R. (2011). Callose biosynthesis regulates symplastic trafficking during root development. *Dev. Cell* 21 1144–1155. 10.1016/j.devcel.2011.10.006 22172675

[B128] Vergara-Silva F. Martínez-Castilla L. Alvarez-Buylla E. R. (2000). MADS-box genes: development and evolution of plant body plans. *J. Phycol.* 36 803–812. 10.1046/j.1529-8817.2000.03654.x

[B129] Wayne R. (2009). *Plant Cell Biology.* Amsterdam: Elsevier.

[B130] Xoconostle-Cazares B. Yu X. Ruiz-Medrano R. Wang H. L. Monzer J. Yoo B.-C. (1999). Plant paralog to viral movement protein that potentiates transport of mRNA into the phloem. *Science* 283 94–98. 10.1126/science.283.5398.94 9872750

[B131] Yruela I. Oldfield C. J. Niklas K. J. Dunker A. K. (2017). Evidence for a strong correlation between transcription factor protein disorder and organismic complexity. *Genome Biol. Evol.* 9 1248–1265. 10.1093/gbe/evx073 28430951 PMC5434936

[B132] Zažímalová E. Murphy A. S. Yang H. Hoyerová K. Hošek P. (2010). Auxin transporters—why so many? *Cold Spring Harb. Perspect. Biol.* 2:a001552. 10.1101/cshperspect.a001552 20300209 PMC2829953

[B133] Zhang X. Facette M. Humphries J. A. Shen Z. Park Y. Sutimantanapi D. (2012). Identification of PAN2 by quantitative proteomics as a leucine-rich repeat-receptor-like kinase acting upstream of PAN1 to polarize cell division in maize. *Plant Cell* 24 4577–4589. 10.1105/tpc.112.104125 23175742 PMC3531853

